# E-CAI: a novel server to estimate an expected value of Codon Adaptation Index (eCAI)

**DOI:** 10.1186/1471-2105-9-65

**Published:** 2008-01-29

**Authors:** Pere Puigbò, Ignacio G Bravo, Santiago Garcia-Vallvé

**Affiliations:** 1 Evolutionary Genomics Group, Department of Biochemistry and Biotechnology, Rovira i Virgili University (URV), Campus Sescelades, c/Marcelli Domingo s/n, 43007 Tarragona, Spain; 2 Experimental Molecular Evolution. Institute for Evolution and Biodiversity. University of Muenster, Germany; 3 Infection and cancer, Deutsches Krebsforschungszentrum, Heidelberg, Germany

## Abstract

**Background:**

The Codon Adaptation Index (CAI) is a measure of the synonymous codon usage bias for a DNA or RNA sequence. It quantifies the similarity between the synonymous codon usage of a gene and the synonymous codon frequency of a reference set. Extreme values in the nucleotide or in the amino acid composition have a large impact on differential preference for synonymous codons. It is thence essential to define the limits for the expected value of CAI on the basis of sequence composition in order to properly interpret the CAI and provide statistical support to CAI analyses. Though several freely available programs calculate the CAI for a given DNA sequence, none of them corrects for compositional biases or provides confidence intervals for CAI values.

**Results:**

The E-CAI server, available at , is a web-application that calculates an expected value of CAI for a set of query sequences by generating random sequences with G+C and amino acid content similar to those of the input. An executable file, a tutorial, a Frequently Asked Questions (FAQ) section and several examples are also available. To exemplify the use of the E-CAI server, we have analysed the codon adaptation of human mitochondrial genes that codify a subunit of the mitochondrial respiratory chain (excluding those genes that lack a prokaryotic orthologue) and are encoded in the nuclear genome. It is assumed that these genes were transferred from the proto-mitochondrial to the nuclear genome and that its codon usage was then ameliorated.

**Conclusion:**

The E-CAI server provides a direct threshold value for discerning whether the differences in CAI are statistically significant or whether they are merely artifacts that arise from internal biases in the G+C composition and/or amino acid composition of the query sequences.

## Background

The Codon Adaptation Index (CAI), introduced by Sharp and Li [1], is a measure of the synonymous codon usage bias for a DNA or RNA sequence and measures the resemblance between the synonymous codon usage of a gene and the synonymous codon frequencies of a reference set. The CAI index ranges from zero to one being one if a gene always uses, for each encoded amino acid, the most frequently used synonymous codon in the reference set. Though it was originally developed to assess how effective selection has been at moulding the pattern of codon usage [1], it has since been applied to problems such as predicting the expression level of a gene [2], predicting a group of highly expressed genes [3,4], assessing the adaptation of viral genes to their hosts [1], giving an approximate indication of the likely success of heterologous gene expression [5], making comparisons of codon usage preferences in different organisms [1], identifying horizontally transferred genes [6-8], detecting dominating synonymous genomic codon usage bias in genomes [9], acquiring new knowledge about species lifestyle [10], and identifying the causes of protein rate variation [11,12].

Since the absolute value of the CAI depends on the query sequence and on the reference set, both of these parameters are important for correctly interpreting CAI values. On the one hand, if the reference set has a random synonymous codon usage with few differences in the use of synonymous codons, the CAI values will be high, i.e. close to one. On the other hand, extreme G+C and/or amino acid compositions on the query sequence may lead to extreme CAI values that are not directly linked to codon usage preferences. It is therefore essential to define a threshold level for the expected CAI value (eCAI) in order to interpret the significance of codon usage biases and to provide statistical support to CAI analyses. The eCAI estimated by our server makes it possible to discern whether differences in the CAI are statistically significant or whether they cannot be distinguished from biases due to nucleotide or amino acid composition. Although several authors have used some kind of expected codon usage [13,14], there is no server or program available to estimate it.

## Implementation

The E-CAI server uses a novel algorithm that calculates an expected CAI for a set of query sequences by generating random sequences with similar G+C content and amino acid composition to the query sequences. The server, implemented in PHP, is integrated with several tools for the calculation and graphical representation of CAI. CAI value is calculated as Sharp and Li originally defined it [1] but using the recent computer implementation proposed by Xia [15]. The Perl source code and a graphical interface written in Tcl/Tk, as well as a tutorial, a Frequently Asked Questions (FAQ) section and several examples are available on the server homepage.

### Inputs of the server

The basic inputs for calculating the expected CAI value are the query sequences, the codon usage of the reference set and the genetic code used. The query sequences must be DNA or RNA sequences in fasta format. The codon usage of the reference set can be introduced in a variety of formats, including the format of the Codon Usage Database [16]. Optionally, the user can introduce a G+C percentage to generate the random sequences. If this G+C percentage is not introduced, the server uses the G+C percentage from the query sequences.

### Generation of the random sequences and estimation of the expected CAI

The method for estimating an expected CAI is based on generating 500 random sequences with the same amino acid composition as the query but with codon usage assigned randomly, either on the basis of the average G+C content of the input, or on the basis of the G+C percentage introduced by the user. Once all random sequences are generated, their CAI values are calculated. The normality of the CAI values of the random generated sequences is assessed with a Kolmogorov-Smirnov Test. An expected CAI value is then estimated using an upper one-sided tolerance interval for a normal distribution and a confidence limit and a percentage of the population (also called coverage) chosen by the user [17]. A tolerance interval is a way to determine a range within which, with some confidence, a specified proportion of a population falls. The eCAI therefore represents the upper limit of the CAI for sequences with a codon usage caused solely by mutational bias. This means that if the CAI value of a gene is bigger than the expected value estimated on composition bias alone, it may be considered evidence of codon usage adaptation or selection. An effective and intuitive way to compare the CAI value of a gene with its expected CAI value is to use that we call the normalised CAI value. This normalised CAI is defined as the quotient between the CAI of a gene and its expected value eCAI.

The E-CAI server allows two methods for generating the random sequences. The first one, called *Markov*, is a Markov Model of order 0. This means that the probability of finding an amino acid at a specific position is independent of the other amino acid positions. The Markov method generates the random sequences by adding one amino acid each time, using the frequencies of each amino acid in the query sequences and a random number. It chooses a random number in the interval (0,1), sums the fractions of the amino acid composition of the query and assigns as the next amino acid the one that causes the sum to exceed the random number [18]. This process is repeated until the desired length of the sequence is reached. The random sequences are then back-translated to DNA sequences, assigning randomly one of the synonymous codon to each amino acid, either on the basis of the average G+C content of the input or on the basis of the G+C percentage introduced by the user. The second method for generating the random sequences, called *Poisson*, is based on the assumption that the number of occurrences for each amino acid in a sequence follows a Poisson distribution. The normalised amino acid frequencies in the query sequences multiplied by the length (*n*) of the generated random sequences are used as the expected numbers of occurrences of each amino acid in the random sequences. These values are used to calculate the probabilities that there were exactly *k *occurrences of each amino acid in a sequence of length *n*. From the sum of these probabilities and a random number, the expected number of occurrences for each amino acid in a random sequence is calculated in a similar way to the Markov method. This process is repeated until the desired number of sequences has been generated. Again, the random sequences are then back-translated to DNA sequences by the same method described above. The results generated by the Markov and Poisson methods are comparable, but the Markov method is more precise and the Poisson method is faster. In addition, similar values of eCAI are obtained when the GenRGenS software is used to generate the random sequences [19].

### Interpretation of the results

The reference set used to calculate the CAI is important for the correct interpretation of its meaning. The CAI measures the similarity between the synonymous codon usage of a gene and the synonymous codon frequency of a reference set. If this reference set is a group of highly expressed genes and in the presence of selected codon usage bias, the CAI values can be used to predict the expression level of genes [20]. However, there is an intrinsic weakness in the interpretation of CAI values when used for species with a highly biased base composition [21]. A further problem also may arise when CAI is used in species which do not display a dominant translational bias [9,20]. Therefore, it is necessary to establish whether highly expressed genes have translationally selected biased codon usage [20]. In this respect, the algorithm E-CAI can successfully overcome the effects of compositional biases when calculating CAI values. If the average codon usage of a genome is used as a reference set, the CAI can be interpreted as a measure of the codon adaptation of a gene in the context of a genome. This information can be used to optimise the expression of a gene in a heterologous expression system [5]. The values of eCAI calculated by the E-CAI server are expected to be over-estimations because the synonymous codon usage of genes is highly influenced by the G+C content at the third codon position and because amino acid usage is also species-specific [22]. The query sequences define both nucleotide and amino acid composition and are therefore important factors in the calculation of eCAI. The expected CAI value could be meaningless if the composition of the query sequences are very heterogeneous. To assess the homogeneity of the sequences in the query set, a Chi-Square test is calculated to test the goodness-of-fit between the amino acid composition or G+C content of each of the query sequences and the average values used to generate the random sequences. The percentage of query sequences that fit the amino acid and/or G+C mean distributions are then shown. If the query sequences are compositionally very heterogeneous, these percentages will be small. In this case we suggest splitting the query sequences into smaller and homogeneous subsets and estimating the eCAI values for each of the subsets separately.

### Executable version

To calculate CAI values for hundreds or thousands of sequences on a whole-genome scale and generate an eCAI, users can download an executable program that automatically performs these calculations. The inputs, methods and outputs of this executable version are the same as those of the web version. However, it enables to choose the length and number of randomly generated sequences. More details about this script and how to use it are found in the tutorial.

## Results

### Example: The Amelioration of mitochondrial genes encoded in the human nuclear genome

It is widely accepted that mitochondria have their origin in a single event, arising from a bacterial symbiont whose closest contemporary relatives are found within the alfa-proteobacteria [23,24]. Since its origin, the mitochondrial genome has undergone a streamlining process of genome reduction with intense periods of loss of genes [25]. Nowadays, mitochondrial genomes exhibit a great variation in protein gene content among most major groups of eukaryotes, but only limited variation within large and ancient groups. This suggests a very episodic, punctuated pattern of mitochondrial gene loss over the broad sweep of eukaryotic evolution [26]. Mitochondrial genomes have lost genes that lack a selective pressure for their conservation. This could include genes whose function may no longer be necessary, genes whose function has been superseded by some pre-existing nuclear genes or genes that were originally present in the proto-mitochondria and that have been transferred to the nucleus [25]. The gene content of present mitochondrial genomes varies from 63 protein-coding genes in *Reclinomonas americana*, a flagellate protozoon, to three genes in other species (see the GOBASE database [27], which contains information for more than 1500 complete mitochondrial genomes). Mitochondria in vertebrates encode for 13 respiratory-chain proteins and for a minimal set of tRNAs that suffices to translate all codons. However, the vast majority of proteins located in the mitochondria are the product of nuclear genes. These genes are encoded and transcribed in the nucleus, translated in the cytoplasm and the proteins are subsequently vehiculated to the mitochondria. Some of these proteins are orthologous of present prokaryote genes and are thought to be the result of horizontal gene transfer events from the proto-mitochondrial to the nuclear genome. This hypothesis is reinforced by the fact that several of these genes are encoded in the mitochondrial genome in other eukaryotic species [28].

To exemplify the use of the CAI server and the significance of expected CAI values, we have analyzed the differential codon adaptation of human mitochondrial genes to both the human codon usage and the mitochondrial codon usage. We used the human codon usage table from Lander et al. [29] and the mean codon usage of all genes from human mitochondrial genome (GenBank accession number AF347015) as human and mitochondrial reference sets, respectively. We have focused on genes that encode for a subunit of the mitochondrial respiratory chain complexes I to V, excluding those that lack a prokaryotic orthologue. Finally, we have divided the genes into two categories according to whether they are encoded in the nuclear or in the mitochondrial genome. Our results are summarised in Table 1, which shows the CAI values with respect to human codon usage (CAIhm) and to the average codon usage of genes encoded in the human mitochondrial genome (CAImt). More than half of the analyzed nuclear-encoded mitochondrial genes from human are present in the mitochondrial genome in other organisms, thus reflecting their proto-mitochondrial origin. Because of the heterogeneity in G+C content of the mitochondrial genes encoded in the nucleus, an expected value (eCAI) was estimated individually for each gene using the Poisson method, a 95% level of confidence and 99% coverage. These expected values are also shown in Table 1, as is the normalised CAI value, which is defined as the quotient between the CAI for each gene and its expected value. A value greater than one in this normalised expected CAI value means that the observed CAI is bigger than its expected value, which could be interpreted as the result of an adaptation process in the codon usage. Table 1 shows that most nuclear-encoded mitochondrial genes are better adapted to the nuclear codon usage than what would be expected by chance, while mitochondrial-encoded mitochondrial genes are better adapted to the mitochondrial codon usage than what would be expected by chance. The CAIhm values of all thirteen mitochondrial-encoded mitochondrial genes are below their expected upper limit, estimated using a sample of random genes with the same G+C content and amino acid composition (Table 1b). At the same time, twelve out of these thirteen genes have a CAImt above their expected upper limit at a 99% confidence level and 95% coverage. The obvious interpretation, therefore, is that mitochondrial-encoded mitochondrial genes are better adapted to mitochondrial codon usage than to nuclear codon usage. Conversely, nuclear-encoded mitochondrial genes are better adapted to nuclear codon usage than to mitochondrial codon usage. Within nuclear-encoded mitochondrial, 34 out of 37 genes show a CAIhm above the expected upper limit at a 95% confidence level and 99% coverage, whereas only two genes have a CAImt above the expected upper limit at a 95% confidence level and 99% of coverage (Table 1a). We interpret this result so that the codon usage of the genes originally encoded in the proto-mitochondria and that are now encoded in the human nuclear genome has been ameliorated and adapted to the human codon usage after their transfer to the nucleus. The E-CAI server provides individual CAI values for each gene with respect to both the nuclear and mitochondrial codon usages, as well as independent eCAI threshold values for differentiating true codon usage optimization from spurious random matches that may arise from compositional biases.

**Table 1:** Analysis of human mitochondrial genes that encode a subunit of complexes I-V of the mitochondrial respiratory chain encoded in the nuclear (a) or mitochondrial (b) genome.

**a) Nuclear encoded genes**										
**Complex**	**Gene name**	**Length**	**CAI_hm_**		**eCAI_hm_**	**CAI_hm_/eCAI_hm_**	**CAI_mt_**		**eCAI_mt_**	**CAI_mt_/eCAI_mt_**
								
					**p = 0.05**	**p = 0.05**			**p = 0.05**	**p = 0.05**

**I**	NDUFS1	2184	0.695	*	0.683	**1.018**	0.434		0.519	0.836
	NDUFS2	1392	0.765	**	0.734	**1.042**	0.391		0.500	0.782
	NDUFS3	795	0.754	*	0.750	**1.005**	0.402		0.488	0.824
	NDUFS7	642	0.867	**	0.780	**1.112**	0.442		0.446	0.991
	NDUFS8	633	0.868	**	0.796	**1.090**	0.439		0.465	0.944
	NDUFV1	1395	0.825	**	0.774	**1.066**	0.417		0.482	0.865
	NDUFV2	750	0.695		0.703	0.989	0.449		0.519	0.865

**II**	SDHC	510	0.699	*	0.679	**1.029**	0.377		0.457	0.825
	SDHD	480	0.663	*	0.654	**1.014**	0.387		0.464	0.834
	SDHA	1995	0.768	*	0.750	**1.024**	0.423		0.496	0.853
	SDHB	843	0.778	**	0.754	**1.032**	0.454		0.481	0.944

**III**	UQCRFS1	825	0.711	*	0.711	**1.000**	0.391		0.483	0.810
	CYC1	978	0.759	*	0.750	**1.012**	0.379		0.449	0.844

**IV**	COX10	1332	0.744	**	0.713	**1.043**	0.454		0.462	0.983
	COX11	831	0.738	*	0.725	**1.018**	0.407		0.513	0.793
	COX15	1140	0.707	*	0.688	**1.028**	0.411		0.472	0.871

**V**	ATP5B	1590	0.714	*	0.698	**1.023**	0.412		0.507	0.813
	ATP5A1	1512	0.695	*	0.684	**1.016**	0.409		0.519	0.788
	ATP5C1	897	0.726	*	0.705	**1.030**	0.463		0.509	0.910
	ATP5O	642	0.700	**	0.681	**1.028**	0.429		0.486	0.883
	ATP5D	507	0.807	**	0.748	**1.079**	0.410		0.426	0.962
	ATP5G1	411	0.776	**	0.707	**1.098**	0.456		0.482	0.946
	ATP5G2	474	0.752	**	0.686	**1.096**	0.472	*	0.451	**1.047**
	ATP5G3	429	0.720	**	0.678	**1.062**	0.430		0.510	0.843
	ATP6V1A	1854	0.709	*	0.702	**1.010**	0.451		0.525	0.859
	ATP6V1B1	1536	0.703		0.711	0.989	0.439		0.514	0.854
	ATP6V1D	744	0.676		0.697	0.970	0.430		0.522	0.824
	ATP6V1E1	681	0.721	*	0.713	**1.011**	0.431		0.500	0.862
	ATP6V1E2	681	0.777	**	0.733	**1.060**	0.410		0.466	0.880
	TCIRG1	2493	0.857	**	0.781	**1.097**	0.421		0.434	0.970
	ATP6V0D2	1053	0.732	*	0.722	**1.014**	0.456		0.518	0.880
	ATP6V0C	468	0.838	**	0.748	**1.120**	0.511	**	0.461	**1.108**
	ATP6F	618	0.803	**	0.741	**1.084**	0.510		0.514	0.992
	ATP6V0D1	1056	0.831	**	0.793	**1.048**	0.457		0.495	0.923
	ATP6V0A1	2496	0.758	*	0.734	**1.033**	0.424		0.507	0.836
	ATP6V0A4	2523	0.770	**	0.735	**1.048**	0.458		0.494	0.927
	ATP6V0A2	2571	0.748	*	0.728	**1.027**	0.450		0.491	0.916

**b) Mitochondrial encoded genes**										

**Complex**	**Gene Name**	**Length**	**CAI_hm_**		**eCAI_hm_**	**CAI_hm_/eCAI_hm_**	**CAI_mt_**		**eCAI_mt_**	**CAI_mt_/eCAI_mt_**

					**p = 0.05**	**p = 0.05**			**p = 0.05**	**p = 0.05**

**I**	ND1	957	0.635		0.796	0.798	0.760	**	0.456	**1.667**
	ND2	1044	0.616		0.774	0.796	0.677	**	0.457	**1.481**
	ND3	345	0.571		0.703	0.812	0.701	**	0.461	**1.521**
	ND4L	297	0.550		0.679	0.810	0.738	**	0.472	**1.564**
	ND4	1377	0.612		0.654	0.936	0.722	**	0.455	**1.587**
	ND5	1812	0.651		0.750	0.868	0.723	**	0.471	**1.535**
	ND6	525	0.612		0.754	0.812	0.361		0.551	0.655

**III**	CYTB	1134	0.655		0.711	0.921	0.758	**	0.481	**1.576**

**IV**	COX1	1542	0.644		0.750	0.859	0.715	**	0.509	**1.405**

	COX2	684	0.641		0.713	0.899	0.664	**	0.503	**1.320**
	COX3	780	0.656		0.725	0.905	0.704	**	0.497	**1.416**

**V**	ATP8	207	0.606		0.688	0.881	0.633	**	0.452	**1.400**
	ATP6	681	0.629		0.698	0.901	0.701	**	0.472	**1.485**

Expected CAIs (eCAIs) at 95% (p = 0.05) and 99% (p = 0.01) confidence and 99% coverage were calculated using the Poisson method of the E-CAI server. For the sake of clarity, only the eCAI values at p = 0.05 are shown. CAIhm and CAImt mean CAI calculated using the mean nuclear and mitochondrial codon usage as a reference set, respectively. CAI values were calculated using the CAIcal tool .

* and ** mean that the CAI is higher than the eCAI estimated at 95% (*) and 99% (**) confidence and 99% coverage. Normalised CAI values (defined as the quotient between the CAI and its expected value) bigger than one are in bold and must be interpreted as evidence of adaptation to the reference codon usage beyond mere compositional biases.

**Figure 1:**
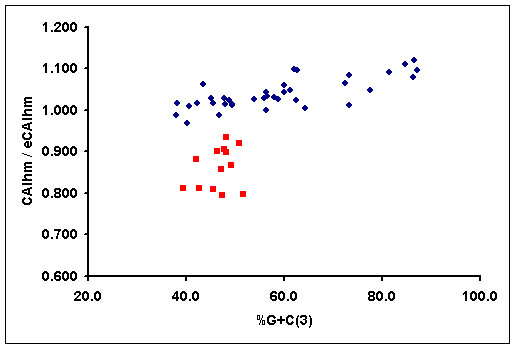
**Graphical representation of the normalised CAIhm, defined as the quotient between the CAI of a gene and its expected value, versus G+C content at the third codon positions for the human genes that encode a subunit of a complex of the mitochondrial respiratory chain**. Red squares represent mitochondrial genes encoded in the human mitochondrial genome and blue dots represent mitochondrial genes encoded in the human nuclear genome. An expected value of CAI was estimated for each gene with the E-CAI server, using the Poisson method and a 95% interval confidence and a 99% population coverage.

Several nuclear-encoded mitochondrial genes have a higher G+C content than mitochondrial-encoded mitochondrial ones. It could therefore be argued that the differences between CAI values of mitochondrial genes of different origin probably reflect differences in G+C content rather than differences in codon usage adaptation. To address this issue, in Figure 1 we have represented the normalised CAIhm of human mitochondrial genes against their G+C content at third codon position. Although some mitochondrial genes encoded in the nuclear genome have a higher G+C content than mitochondrial encoded ones, there are several mitochondrial genes, encoded in the nuclear and mitochondrial genome, with similar G+C contents. However, the normalised CAIhm is very different in both populations (figure 1), as is also demonstrated if a Kolmogorov-Smirnoff test (D = 1.0, P < 0.0001) is used. This clearly shows that the codon usage of the nuclear encoded genes is not only due to mutational pressure or G+C content, and that a certain degree of codon usage adaptation exists. In this sense, it has recently been reported that a weak positive correlation between gene expression levels and the frequency of optimal codons exists in humans [30,31].

## Conclusion

The E-CAI server described here provides an expected value of CAI for discerning whether the differences in CAI are statistically significant and arise from the codon preferences or whether they are merely artifacts that arise from internal biases in the G+C composition and/or amino acid composition of the query sequences. Using a normalised CAI value, defined as the quotient between the CAI of a gene and its expected value, is an effective and intuitive way to analyze the codon usage bias of genes and codon usage adaptation.

## Availability and requirements

• **Project name: **E-CAI

• **Project home page: **

• **Operating system(s): **Platform independent

• **Programming language: **PHP

• **Other requirements: **none

• **Any restrictions to use by non-academics: **license needed

## Authors' contributions

PP designed the server, made the programming task, helped draft the manuscript and prepared the example. IGB participated in design of the server, developed the Poisson-based method, and helped draft the manuscript. SG-V conceived and designed the server, coordinated the project and drafted the manuscript. All authors read and approved the final manuscript.
